# Design of Liposome Formulations for CRISPR/Cas9 Enzyme Immobilization: Evaluation of 5-Alpha-Reductase Enzyme Knockout for Androgenic Disorders

**DOI:** 10.1021/acsomega.3c07138

**Published:** 2023-11-20

**Authors:** Hasan Akbaba, Gülşah Erel-Akbaba, Yücel Başpınar, Şerif Şentürk

**Affiliations:** † Department of Pharmaceutical Biotechnology, Faculty of Pharmacy, Ege University, Izmir 35100, Turkey; ‡ Department of Pharmaceutical Biotechnology, Faculty of Pharmacy, Izmir Katip Celebi University, Izmir 35620, Turkey; § Izmir Biomedicine & Genome Center, Izmir 35340, Turkey; ∥ Genome Sciences & Molecular Biotechnology, Izmir International Biomedicine & Genome Institute, Dokuz Eylul University, Izmir 35340, Turkey

## Abstract

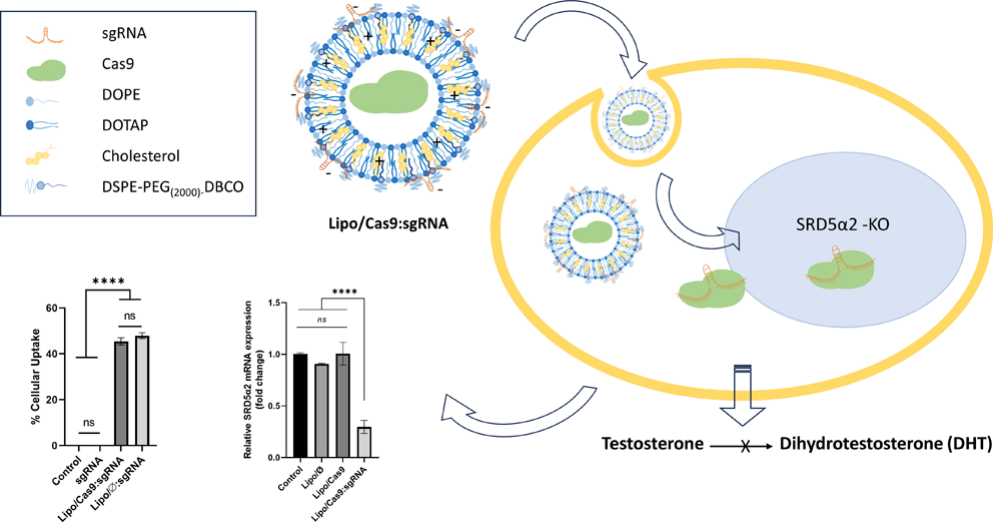

The enzyme steroid type II 5-alpha-reductase (SRD5α2) is responsible for the conversion of testosterone to dihydrotestosterone (DHT), which is involved in prostate cancer, benign prostatic hyperplasia, and androgenic alopecia. Inhibition of SRD5α2 activity has been explored and presented as a potential treatment for these conditions, but current drugs have side effects and alternative treatment approaches are needed. The CRISPR/Cas9 system, an innovative gene-editing tool, shows potential for targeting the SRD5α2 gene knockout as a therapeutic approach. Liposomes have been used for the immobilization and delivery of different proteins, and studies have shown that liposomes can enhance the stability and activity of enzymes. In this study, we provided the immobilization of Cas9 protein by encapsulating it in a novel cationic liposome formulation that carries sgRNA on its outer surface for gene delivery approaches. This novel delivery system has shown promising results in terms of physicochemical properties, stability, cytotoxicity, *in vitro* cellular uptake, and gene knockout efficiency, together with providing flexibility in sgRNA selection. The optimized final formulations showed an average diameter of 229.1 ± 3.66 nm, a polydispersity index of 0.089 ± 0.013, and a zeta potential value of 25.7 ± 0.87 mV. The encapsulation efficiency of the developed formulations has been revealed as 80.60%. The cellular uptake efficiency was evaluated and measured as 45.6% for the final formulation. Furthermore, the Lipo/Cas9:sgRNA (1.5:1) formulation decreased the relative SRD5α2 mRNA expression by 29.7% compared to the control group. The results of this study reveal that the liposomal formulation based on enzyme immobilization of Cas9 protein using CRISPR technology, an innovative gene-editing tool for SRD5α2 suppression, might be an alternative treatment option for prostate cancer or BPH treatment without current drug side effects.

## 1 Introduction

Steroid type II 5-alpha-reductase (SRD5α2) is an enzyme that plays a crucial role in the conversion of testosterone to dihydrotestosterone (DHT), the most potent androgen in the prostate gland.^1,2^ The enzyme is encoded by the SRD5α2 gene, which is responsible for male pseudo hermaphroditism due to 5-alpha-reductase deficiency, as well as being involved in androgen-related diseases such as prostate cancer (PC) and benign prostatic hyperplasia (BPH). The SRD5α2 activity is crucial for the normal development of the external genitalia and prostate in human males.^3^ Studies have shown that 5-alpha-reductase activity is higher in BPH tissue and PC cell lines than in normal prostate tissue.^4^ Abnormally high 5-alpha-reductase activity in humans produces excessively high DHT levels in peripheral tissues, which is implicated in the pathogenesis of PC and BPH.^5,6^ Therefore, the inhibition of 5-alpha-reductase activity has been explored as a potential treatment for these conditions.

Several drugs have been developed to inhibit 5-alpha-reductase activity, including finasteride and dutasteride.^1^ These drugs have been used to treat BPH and male pattern hair loss and have also been investigated for their potential to reduce the risk of PC.^7^ Inhibition of 5-alpha-reductase activity has been explored as a potential treatment for these conditions, but the use of currently prescribed drugs has been associated with adverse effects such as gynecomastia, depression, erectile dysfunction, and diminished libido.^8^ Further research is needed to develop a more effective and safer way to decrease the level of SRD5α2 for the treatment of these conditions.

Genome editing using the CRISPR (clustered regularly interspaced short palindromic repeats)/Cas9 (CRISPR-associated protein 9) system has revolutionized the field of biomedicine, offering new approaches to understanding and treating diseases. The Cas9 enzyme, an RNA-guided DNA endonuclease, is a critical component of this system and has been widely used in various areas of biomedical research. The high efficiency and accuracy of the CRISPR/Cas9 system make it a promising treatment strategy.^9^ However, the clinical translation of CRISPR/Cas9-mediated genome editing for disease treatment is still in its early stages, and several challenges need to be addressed, such as off-target effects, delivery methods, and ethical considerations.^10−13^ CRISPR/Cas9-mediated genome editing holds great potential for developing new treatments for various human genetic diseases, and further research is needed to overcome the existing challenges and ensure the safety and efficacy of this technology.

The CRISPR/Cas9 system can knock out the SRD5α2 gene as a therapeutic approach against PC and BPH.^14^ The use of 5-alpha-reductase inhibitors, such as finasteride and dutasteride, is a standard treatment for BPH, and it has been reported to improve BPH-related outcomes and reduce the detection of PC.^15−17^*In vitro* experiments have shown that the use of 5-alpha-reductase inhibitors could be effective in reducing the risk of PC and be a promising treatment for PC.^17,18^ However, safety concerns are associated with using 5-alpha-reductase inhibitors, including an increased risk of cardiac failure and sexual dysfunction.^7,19^ Therefore, the use of the CRISPR/Cas9 system to knock out the 5-alpha-reductase enzyme in the treatment of PC and BPH requires the potential to overcome these obstacles.

Enzyme immobilization is a process in which enzymes are attached to a delivery system, resulting in enhanced reusability, stability, and catalytic activity. The advantages of enzyme immobilization include the elimination of enzyme isolation and purification steps, reduction in the volume of bioreactors, and increased stability toward environmental conditions such as pH, temperature, ionic strength, and cost-effectiveness.^20−22^ Enzyme immobilization using liposome formulations is a promising strategy for enhancing enzyme stability, activity, and reusability. Liposomes are spherical vesicles composed of a phospholipid bilayer that can encapsulate enzymes, protect them from harsh environments, and take advantage of biomembrane functions.^23−25^

Several studies have investigated the liposome formulations of plasmid-based Cas9 or protein-based Cas9 complexed with a single guide RNA (sgRNA)—heterogeneous nuclear ribonucleoproteins (hnRNPs)—for higher gene editing efficiency.^26−28^ However, there is no study in the literature in which Cas9 protein was immobilized without sgRNA, and its effectiveness was investigated to provide high gene regulation and functionality.

In this study, we provided the immobilization of the Cas9 protein by encapsulating it in a liposome formulation. We have shown the possibility of gene editing with obtained liposome formulations carrying sgRNA on their outer surface for gene delivery; thus, researchers gain a broad functionality that can provide an extensive repertoire of sgRNA selection. For this purpose, novel cationic liposomes were prepared and characterized in terms of particle properties like average diameter, polydispersity index, zeta potential and morphology, physical stability, cytotoxicity, complex formation with sgRNA, encapsulation efficiency, loading capacity, serum stability, Cas9 release, and *in vitro* cellular uptake. Our study reveals that liposome formulation based on enzyme immobilization of the Cas9 protein using the CRISPR technology, an innovative gene-editing tool for SRD5α2 suppression, might be an alternative treatment option for PC or BPH treatment without current drug side effects.

## 2 Materials and Methods

### 2.1 Materials

Cholesterol was obtained from Sigma-Aldrich Chemical Co. (St.Louis, USA). 1,2-Dioleoyl-*sn*-glycero-3-phosphoethanolamine (DOPE) and 1,2-distearoyl-*sn*-glycero-3-phosphoethanolamine-*N*-[dibenzocyclooctyl(polyethylene glycol)-2000] (DSPE-PEG_(2000)_-DBCO) were purchased from Avanti Polar Lipids (Alabaster, USA). 1,2-Dioleoyl-3-trimethylammoniumpropane (DOTAP) was provided by Cayman Chemicals (Ann Arbor, USA). L929 and DU145 cells were obtained from the American Type Culture Collection (Virginia, USA). All other chemicals were used as analytical grade. Ultrapure nuclease-free water (UPH_2_O) was used in all stages needed.

The SRD5α2 exon region complemented oligonucleotide sequences verified in accordance with the CRISPR system and determined with web-based sgRNA design tools. sgRNA was synthesized by an *in vitro* transcription method using the EnGen sgRNA Synthesis Kit, *S. pyogenes* (NEB, USA). Accordingly, the template oligo DNA design for the production of sgRNA is as follows and is provided from Eurofins Genomics (Wolverhampton, UK).

SRD5α2 sgRNA-template: TTCTAATACGACTCACTATAGGAGGGCTTCGCGACGTACAGTTTTAGAGCTAGA.

### 2.2 Methods

#### 2.2.1 Preparation of Liposomes

Liposome formulations were prepared by using the film hydration method. Different formulation contents and molar ratios examined are presented in Table 1. DOTAP was used as a cationic agent to impart a positive charge to the formulations. Formulation components were weighed in the specified molar ratios and dissolved in chloroform.^29,30^ A thin lipid film was obtained by removing the solvent at 45 °C with the help of an evaporator for 15 min. Under N_2_ flow, the residual solvent was evaporated. The resulting lipid film was resuspended in 200 mM HEPES buffer (containing 1 M NaCl, 50 mM MgCl_2_, and 1 mM EDTA, pH 6.5), and liposome formulations were obtained.

**Table 1:** Compounds and Their Molar Ratios Are Used to Prepare the Liposomes

formulation code	compound 1	compound 2	compound 3	compound 4	molar ratio
Lipo-1	cholesterol	DOTAP	DOPE	DSPE-PEG_(2000)_-DBCO	1:0.5:0.5:0.1
Lipo-2	cholesterol	DOTAP		DSPE-PEG_(2000)_-DBCO	1:1:0:0.1
Lipo-3		DOTAP	DOPE	DSPE-PEG_(2000)_-DBCO	0:1:1:0.1

The obtained multilamellar vesicles were serially extruded 10 times through membrane filters (Whatman, USA) having 0.8, 0.6, and 0.22 μm pore sizes, respectively.^31^

The obtained empty formulations were evaluated in terms of their characterization properties, and stability monitoring was carried out in order to determine the optimal formulation.

Cas9-loaded liposome formulation (Lipo/Cas9) was prepared by dissolving the lipid film in Cas9 enzyme solution (10 μM). The final lipid concentration of the liposomes was 5 mg/mL.

#### 2.2.2 Characterization and Stability of Liposomes

The resulting liposomes were characterized with respect to the average diameter (AD), polydispersity index (PI), and surface charge (zeta potential, ZP) using the dynamic light scattering technique (DLS, Litesizer 500, Anton Paar, Austria). DLS measurements were performed at 25 °C and reported as mean intensity-weighted distributions of AD measurements.^32,33^

A Carl ZEISS Sigma 300 VP scanning electron microscope (SEM—ZEISS Group, Germany) was used for morphological investigations.^34^ Samples were coated with a conductive gold layer using a He Quorum Q150R ES (Quorum Technologies, UK) under a high vacuum prior to analysis. SEM analysis was then performed at the Central Laboratory of Katip Çelebi University, Izmir, Turkey, using an InLens secondary electron detector operating at 2.00 kV.

The physicochemical stability of liposomes was investigated after storage of 60 days at +4° and −20 °C by measuring the AD, PI, and ZP on days 0, 7, 14, 30, and 60 by a DLS instrument (Litesizer 500, Anton Paar, Austria).

#### 2.2.3 Cytotoxicity Studies

Cytotoxicity studies were performed on the mouse fibroblast cell line L929 and the human PC cell line DU145.^35^ L929 is one of the cell lines to study *in vitro* bioreactivity and cytotoxicity of compounds, delivery systems, and medical devices proposed by the authorities for biological safety evaluation as in the ISO 10993 (Biological evaluation of medical devices) document.^36,37^

DU145 and L929 cells were cultured in a complete medium containing Dulbecco’s modified Eagle’s medium (DMEM, low glucose) with 10% fetal bovine serum (FBS), 2 mM l-glutamine, and 1% penicillin–streptomycin to avoid bacterial contamination. Then, the cells were plated in 96-well plates at a density of 5 × 10^3^ cells per well in 100 μL growth media and treated with increased concentrations (50, 100, 200, 400, 600, and 800 μg/mL based on lipid concentration) of liposome formulations for 24 h. According to the manufacturer’s instructions, the percentage of viable cells was assessed using the Alamar Blue Cell Viability Assay (Thermo Fisher Scientific, USA). Cell viability was calculated by normalizing the fluorescence of the untreated cells. Experiments were performed at least four times.

#### 2.2.4 Encapsulation Efficiency and Loading Capacity of Liposomes

The encapsulation efficiency of Cas9 protein within liposomes was measured using a centrifugal filter system. 100 μL of the liposome suspension was placed in an Amicon Ultra 0.5 centrifuge filter device and centrifuged at 15,000 rpm for 30 min using a Hettich MIKRO 200R centrifuge (Tuttlingen, Germany). The resulting ultrafiltrate was quantified and analyzed using the QuantiPro BCA Assay Kit (Thermo, USA) to determine the concentration of free Cas9.^38^

The encapsulation efficiency was calculated as the percentage of Cas9 protein incorporated into the liposome relative to the total amount of Cas9 used initially according to the following formula
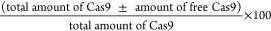


The loading capacity of the liposome samples was also determined. The amount of the Lipo/Cas9 formulation was diluted with 5 mL of ethanol. After sonication and centrifugation, the Cas 9 protein concentration of the supernatant was analyzed using the BCA Assay Kit described above.

The Cas9 loading capacity of the liposome was calculated according to the following formula
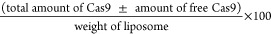


#### 2.2.5 Cas9 Release Studies

*In vitro* drug release experiments were conducted by using Float-A-Lyzer Dialysis Device tubes (100 kDa MW pore size, Sigma-Aldrich, USA). For release studies, 100 μL of Lipo/Cas9 formulation samples were placed in six dialysis kit tubes, each for a different time interval. The release of the encapsulated Cas9 enzyme from the liposome formulation was investigated in Tris–HCL buffer (100-fold volume) at 4 °C and 100 rpm for 0, 1, 3, 6, 12, and 24 h. At the end of each period, the relevant tube was removed from the dialysis medium, and the tube contents were transferred to a separate 1.5 mL sterile microcentrifuge tube. The protein concentration was measured by adding 900 μL of methanol to the liposome system to disrupt its structure and the resulting amount of Cas9 enzyme using the QuantiPro BCA Assay Kit (Thermo, USA). All samples were analyzed three times, and the amount of Cas9 enzyme released was found by subtracting the initial amount of Cas9 enzyme remaining in the tube.

#### 2.2.6 Preparation of Lipo/Cas9:sgRNA Complexes

Based on the stability studies, the optimal liposomal formulation was used for further studies such as liposome-sgRNA complex formation, serum stability, and Cas9 release.

Lipo/Cas9:sgRNA complexes were formed by electrostatic interactions between cationically charged liposomes and anionically charged sgRNAs. To determine the optimal complex formation rate, the cationic lipid molar ratio was increased while the molar ratio of sgRNA remained constant (1.5:1, 2.5:1, 3.75:1, 7.5:1, 15:1, N^+^/P^–^ molar ratio).^39^ For this purpose, the indicated proportion of liposomes was added to the sgRNA solution (110 μg/μL) and the mixture incubated for 30 min at 25 °C on a shaker to complete complex formation. Glycerol (2%) was added to each sample, and the complexes were loaded into the agarose gel wells. Agarose gel electrophoresis (2% agarose in 1 × TAE, w/v) was performed at a constant voltage of 100 V for 50 min. After electrophoresis, gels were stained in a 0.5 μg/mL ethidium bromide solution. The migration pattern of sgRNA was then visualized with a UV transilluminator (GEN-BOX, imagER CFx ER Biotech, Turkey). Naked sgRNA was used as a control.

#### 2.2.7 Serum Stability Assay

Naked sgRNA and Lipo/Cas9:sgRNA complexes were used to assess the serum stability of liposomes.^40,41^ Upon receiving the complexes, active serum was added to the naked sgRNA and Lipo/Cas9:sgRNA complexes at final concentrations of 10 and 50% (v/v) from the 24 h reaction tube. The next day, the reactions were initiated separately for 6, 4, 3, 2, 1, and 0.5 h in each tube, respectively. After adding a serum to the 0 h reaction tubes, proteinase K working solution (200 μg/mL) was added directly to all samples and incubated at 55 °C for 30 min. SDS solution was added to a final concentration of 2% (w/v) to stop the enzymatic activity and release sgRNA from the complex structure. Unthreatened naked sgRNA was used as a control. Agarose gel electrophoresis was performed as described above, and samples were visualized through a UV transilluminator (GEN-BOX, imagER CFx ER Biotech, Turkey). ImageJ software was used to quantify band densities and analyze the degradation effects of serum proteins and nucleases over sgRNA.

#### 2.2.8 *In Vitro* Cellular Uptake Studies

*In vitro* cellular uptake studies were performed on the PC cell line DU145. 24 h before treatment, the cells were seeded in 12-well culture plates at 5 × 10^4^ cells/mL density and incubated overnight. The cells were treated when they reached approximately 60–70% confluency.

For *in vitro* cellular uptake studies, sgRNA was labeled with a fluorescent dye using the Label IT Nucleic Acid Labeling Kit (Mirus Bio, Madison, WI). siRNAs were introduced into cells *via* Lipo/⌀ and Lipo/Cas9 formulations. Equivalent amounts of naked sgRNA were used as controls. Cells were then incubated for 3 h. *In vitro* cellular uptake studies were assessed by both fluorescence microscopy and flow cytometry. First, the cells were imaged by using a fluorescence microscope (IX71, Olympus, Tokyo, Japan). They were then harvested by trypsinization, washed twice with phosphate-buffered saline, suspended in 100 μL of FACs buffer (2 μL of serum in phosphate-buffered saline), and quantitatively analyzed by a flow cytometry device (BD Fortessa, BD Biosciences, San Jose, USA) Untreated cells were included as a negative control. Data were analyzed with FlowJo software V10 (TreeStar, Ashland, DE, USA).

#### 2.2.9 Evaluation of Gene Knockout Efficiency

DU145 cells were precultured in 12-well plates until the cells reached 70–80% confluence (approximately 24 h). Cells were then treated with Lipo/⌀, Lipo/Cas9, Lipo/Cas9:sgRNA1, and Lipo/Cas9:sgRNA2 (400 μg/mL lipoplex, with respect to solid lipids) for 24 h. The cell medium was then replaced with a fresh growth medium and incubated for an additional 24 h. Treated cells were harvested 48 h after treatment, and total RNA was isolated according to the Nucleospin RNA isolation Kit (Macherey-Nagel, Germany) protocol. cDNA templates were then generated by a reverse polymerase reaction using the OneScript Plus cDNA Synthesis Kit (ABM, Richmond, BC, Canada). RT-qPCR was then performed on an AriaMx Real-Time PCR System (Agilent, Santa Clara, CA, USA). Primer pairs (Eurofins, Germany) used to detect SRD5α2 and GAPDH as housekeeping genes are as follows: SRD5α2, 5′-TCA GAC GAA CTC AGT GTA CGG-3′ (forward) and 5′- CGT AGT GGA CGA GGA ACA TGG-3′ (reverse); and GAPDH, 5′ TGT GGG CAT CAA TGG ATT TGG-3′ (forward) and 5′-ACA CCA TGT ATT CCG GGT CAAT-3′ (reverse). Results were analyzed using the ΔΔ*CT* method with untreated cells as controls. Each experiment was repeated at least three times.

#### 2.2.10 Statistical Analysis

GraphPad Prism 8.0 (GraphPad Software, Inc., USA) was used for statistical analysis. Data are expressed as mean ± SD. Statistical analyses between groups were assessed using unpaired *t* tests and one-way ANOVA followed by multiple comparison tests. The difference is considered statistically significant if the calculated *p*-value is less than 0.05.

## 3 Results

**Figure 1:**
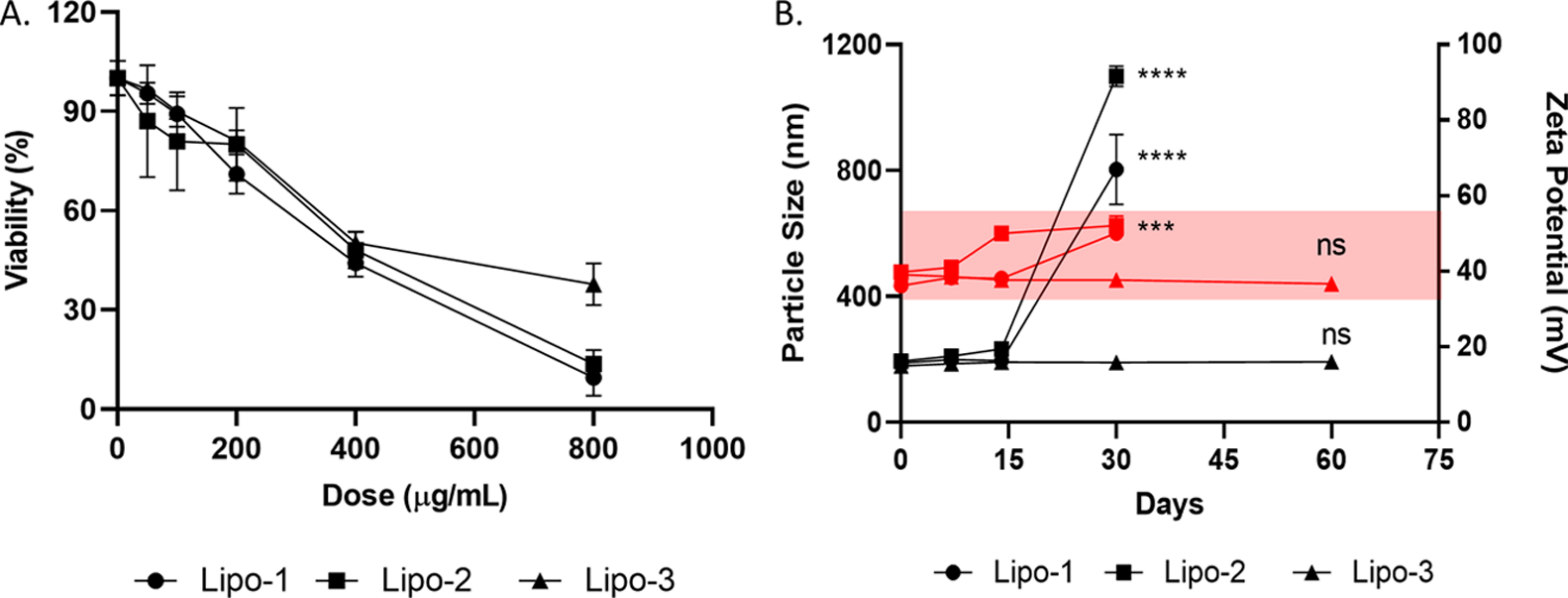
*In vitro* cytotoxicity (A) and physicochemical stability (B) of preformulations Lipo-1/2/3. Data are presented as mean values ± SD of triplicate treatments (*n* = 3). The red area is visualized to highlight the zeta potential results.

Within the scope of preformulation studies, Lipo-1/2/3 formulations without the Cas9 enzyme were produced. Afterward, characterization studies were carried out to determine the formulation with the optimal properties. In addition, the physicochemical stability and *in vitro* bioreactivity of the preformulations were investigated. The AD, PI, and ZP of preformulations were determined by DLS measurements. The AD, ZP, and PI results of the preformulations showed no significant differences (Table 2). Both formulations showed dose-dependent cytotoxicity on L929 mouse fibroblast cells. Lipo-3 showed a significantly lower cytotoxicity for the highest applied dose (Figure 1A). Furthermore, particle physicochemical stability was followed for 2 months, and Lipo-3 showed no significant change in terms of AD and ZP. On the other hand, Lipo-1 and Lipo-3 showed a significant increase in both AD and ZP and were considered to be unstable for further studies (Figure 1B).

**Table 2:** Average Diameter, Polydispersity Index, and Zeta Potential Results of the Prepared Cationic Liposomes^a^

formulation	AD (nm ± SD.)	PI (±SD.)	ZP (mV ± SD.)
Lipo-1	188.19 ± 1.65	0.147 ± 0.03	36.2 ± 0.6
Lipo-2	194.69 ± 5.02	0.206 ± 0.05	39.8 ± 0.4
Lipo-3	179.68 ± 3.40	0.159 ± 0.08	41.7 ± 0.6

a AD = average diameter; PI = polydispersity index; ZP = zeta potential; SD = Standard deviation.

**Figure 2:**
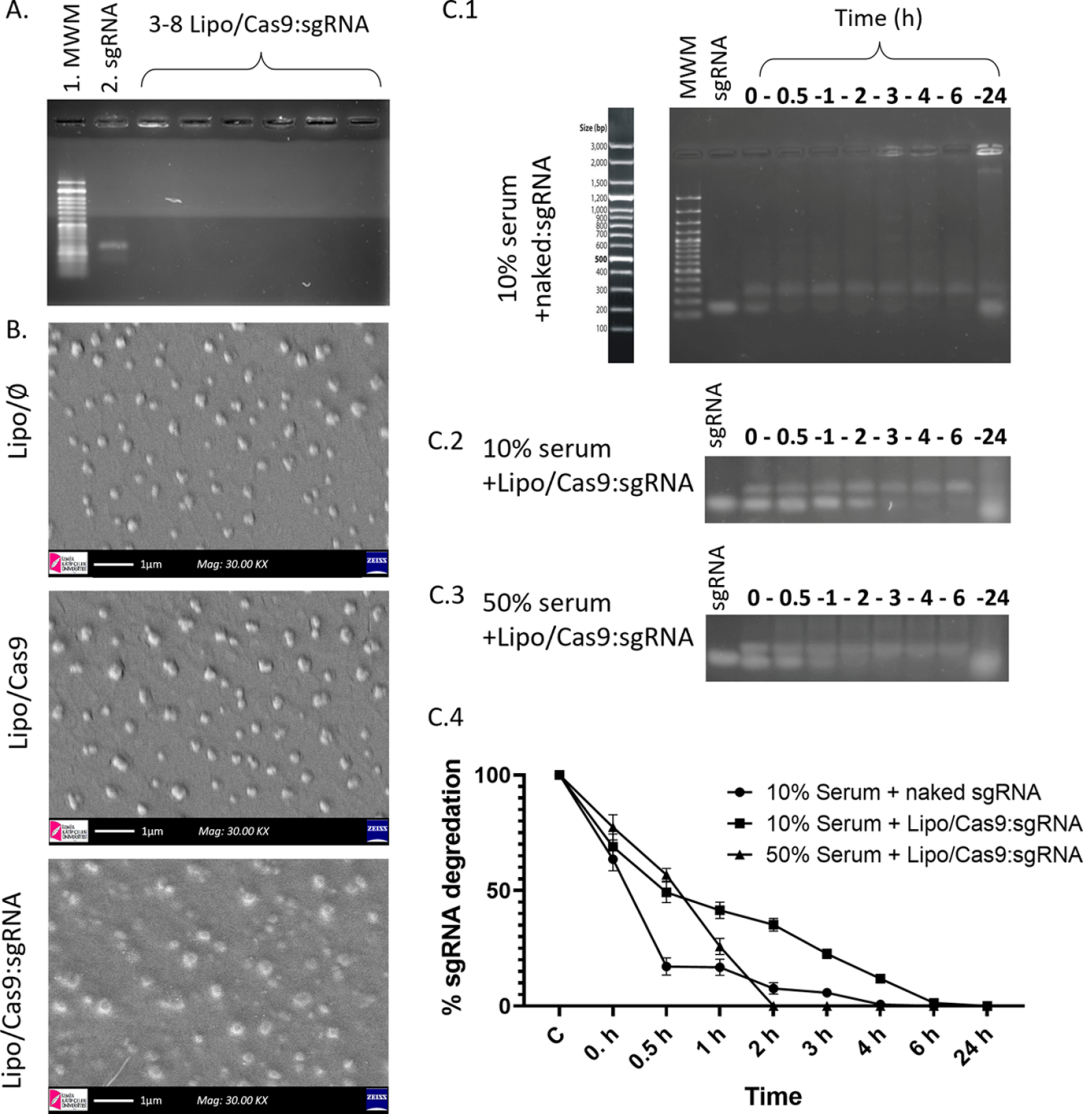
Preparation of Lipo/Cas9:sgRNA complexes and serum stability studies: (A) Agarose gel electrophoresis image of gel retardation assay for Lipo/Cas9:sgRNA complex formation. Lanes from left: 1:100 bp DNA ladder as the molecular weight marker (MWM), 2: naked sgRNA, 3–8: Lipo/Cas9:sgRNA complexes with ratios of 1.5:1, 2.5:1, 3.75:1, 7.5:1, 15:1, and N^+^/P^–^ molar ratio, respectively. (B) SEM images of Lipo/⌀, Lipo/Cas9, and Lipo/Cas9:sgRNA (1.5:1) formulations. (C): Serum stability of sgRNA incubated in active serum at 37 °C for different time points (0, 0.5, 1, 2, 3, 4, 6, and 24 h). Released sgRNA from the Lipo/Cas9:sgRNA complex are shown. Lane 1:100 bp DNA ladder as MWM, lane 2: sgRNA, lanes 3–10: time points (0, 0.5, 1, 2, 3, 4, 6, and 24 h). The density of the gel bands was analyzed for each agarose gel image with ImageJ software using 10% active serum and naked sgRNA (C.1), 10% active serum and Lipo/Cas9:sgRNA (C.2), and 10% active serum and Lipo/Cas9:sgRNA (C.3) and presented graphically (C.4).

Based on the Lipo-3 formulation, the final formulation to be used in Cas9 immobilization was produced. In order to prevent Cas9 from losing its enzyme activity, 200 mM HEPES buffer (contains 1 M NaCl, 50 mM MgCl_2_, 1 mM EDTA, pH 6.5) was used instead of ultrapure water, as in Lipo-3, for the resuspension of the liposome formulation. The formulation containing only buffer solution in the inner phase was produced to be used as a negative control and named as Lipo/⌀. The liposome formulation containing both buffer and a Cas9 enzyme solution (10 μM) was named Lipo/Cas9. In order to determine the ratio of the complex made by Lipo/Cas9 with sgRNA, a gel retardation experiment was carried out, and the optimal complex ratio was determined as 1.5:1 (N^+^/P^–^ molar ratio) as for Lipo/Cas9:sgRNA (sgRNA solution was used as 110 μg/μL concentration) (Figure 2A). Table 3 summarizes the physicochemical characterization results of Lipo/⌀, Lipo/Cas9, and Lipo/Cas9:sgRNA formulations together with the 90 day stability results of Lipo/⌀ and Lipo/Cas9 formulations at 4 °C.

**Table 3:** Average Diameter, Polydispersity Index, and Zeta Potential Results of the Optimized Cationic Liposomes Containing Buffer or Cas9 after Storage of 90 Days at 4 °C^a^

formulation	storage duration (days)	average diameter (nm ± SD.)	PI (±SD.)	ZP (mV ± SD.)
Lipo/⌀	0	166.4 ± 0.52	0.102 ± 0.016	32.5 ± 1.56
	7	169.2 ± 1.93	0.112 ± 0.009	32.2 ± 0.92
	14	166.7 ± 1.71	0.104 ± 0.005	32.3 ± 1.56
	30	168.5 ± 1.47	0.131 ± 0.015	31.0 ± 1.35
	60	181.9 ± 1.54	0.203 ± 0.208	29.5 ± 1.97
	90	184.1 ± 4.05	0.242 ± 0.032	33.1 ± 1.25
Lipo/Cas9	0	190.7 ± 0.56	0.160 ± 0.007	36.6 ± 1.25
	7	187.4 ± 0.81	0.133 ± 0.024	32.9 ± 0.49
	14	189.4 ± 2.52	0.155 ± 0.017	36.0 ± 1.29
	30	202.7 ± 1.65	0.212 ± 0.009	36.2 ± 1.15
	60	435.9 ± 16.7	0.490 ± 0.040	15.7 ± 0.51
	90	1557.1 ± 208.5	0.272 ± 0.087	3.48 ± 1.70
Lipo/Cas9:sgRNA (N^+^/P^–^ molar ratio)	0	229.1 ± 3.66	0.089 ± 0.013	25.7 ± 0.87

a AD = average diameter; PI = polydispersity index; ZP = zeta potential; SD = standard deviation.

It could be shown that the Lipo/Cas9:sgRNA ratio of 1.5:1 (N^+^/P^–^ molar ratio) was needed to bind sgRNA completely to the cationic liposome formulation and block the sgRNA migration (lane 3 or higher).^42^ Thus, this ratio was used to prepare Lipo/Cas9:sgRNA for further studies.

The AD of the Lipo/⌀ formulation slightly increased from 166.4 nm (d0) to 184.1 nm after storage of 90 days at 4 °C, as the ZP very slightly increased from 32.5 to 33.1 mV. However, AD and ZP values did not change statistically significantly (*p* > 0.05). In contrast to that, the PI significantly increased from 0.131 to 0.203 between day 30 to day 60, but is still accepted as monodispersed (*p* = 0.0012). After storage of 30 days at 4 °C, the AD of the Lipo/Cas9 formulation slightly increased from 190.7 to 202.7 nm, as the PI slightly increased from 0.160 to 0.212. The AD, ZP, and PI results of the Lipo/Cas9 showed no significant differences for 30 days and were considered stable with regard to physicochemical characteristics (*p* > 0.05). However, after storage of 60 days at 4 °C, the AD of the Lipo/Cas9 formulation significantly increased to 435.9 nm (*p* < 0.0001), and the ZP significantly decreased from 36.6 to 15.7 mV (*p* < 0.0001), meaning that the Lipo/Cas9 formulation lost its stability at day 60. The liposome formulation containing Cas9 and sgRNA (Lipo/Cas9:sgRNA, 1.5:1 (N^+^/P^–^ molar ratio) showed appropriate properties for cell culture studies like an AD of 229.1 nm, a PI of 0.089, and a ZP of 25.7 mV.

Lipo/⌀, Lipo/Cas9, and Lipo/Cas9:sgRNA formulations’ morphology was visualized using an SEM. Representative SEM photographs revealed that the formulations are nanosized, monodispersed, and within the correlation of DLS measurements (Figure 2B).

The degrading effect of serum proteins and nucleases was monitored for 24 h by a serum stability assay. 10 and 50% serum concentrations were examined. In Figure 2C, Lipo/Cas9:sgRNA (1.5:1) serum stability test results and the naked sgRNA serum stability test results as control are presented. The band densities of both gel images were quantified by ImageJ software. In the optimized protocol, the incubation time of the liposomes in serum-containing full-growth media is 3 h. According to these results, the Lipo/Cas9:sgRNA protected sgRNA up to 3 h at a final serum concentration of 10% and up to 1 h at a final serum concentration of 50%, while naked sgRNA massively degraded in 0.5 h at a 10% serum concentration (Figure 2C).

Encapsulation efficiency (EE) and loading capacity (LC) studies were carried out to determine the Cas9 delivery capacity of the developed Lipo/Cas9. The EE and LC study results of the Lipo/Cas9 formulation revealed an encapsulation efficiency of 80.60% (±1.65) and a loading capacity of 12.59% (±1.01). These results suggest that the optimized liposome formulation is appropriate as a delivery system for Cas9 transcytosis.

**Figure 3:**
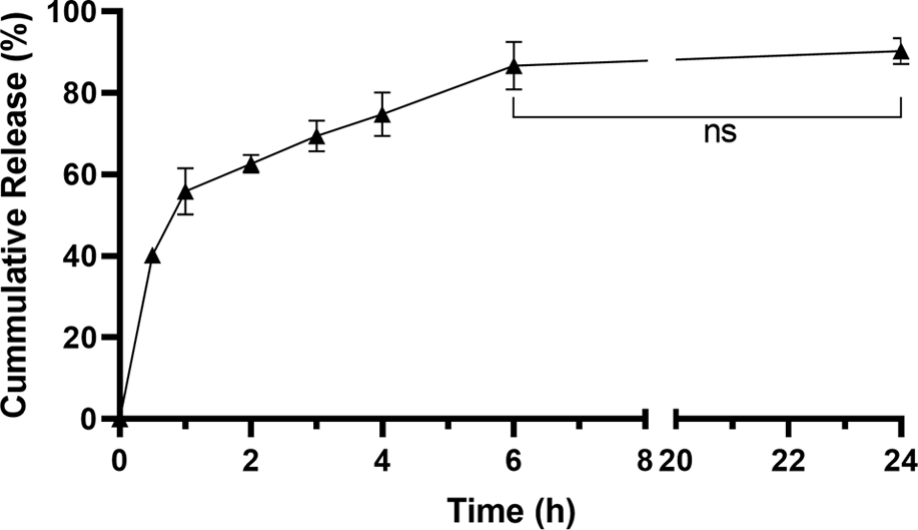
Cumulative *in vitro* release of Cas9 from the Lipo/Cas9 formulation.

After the determination of EE and LC, the release profile of the Lipo/Cas9 was investigated *in vitro* at 37 °C in PBS (pH 7.4). The % cumulative release of Cas9 reached 55.8% after 1 h and 62.6% after 6 h. In 6 h, 86.6% of Cas9 was released from the Lipo/Cas9 formulation, the remaining Cas9 being slowly liberated during the next 24 h, and no significant increase was observed (Figure 3). Thus, the optimized liposome formulation is appropriate as a delivery system for Cas9.

**Figure 4:**
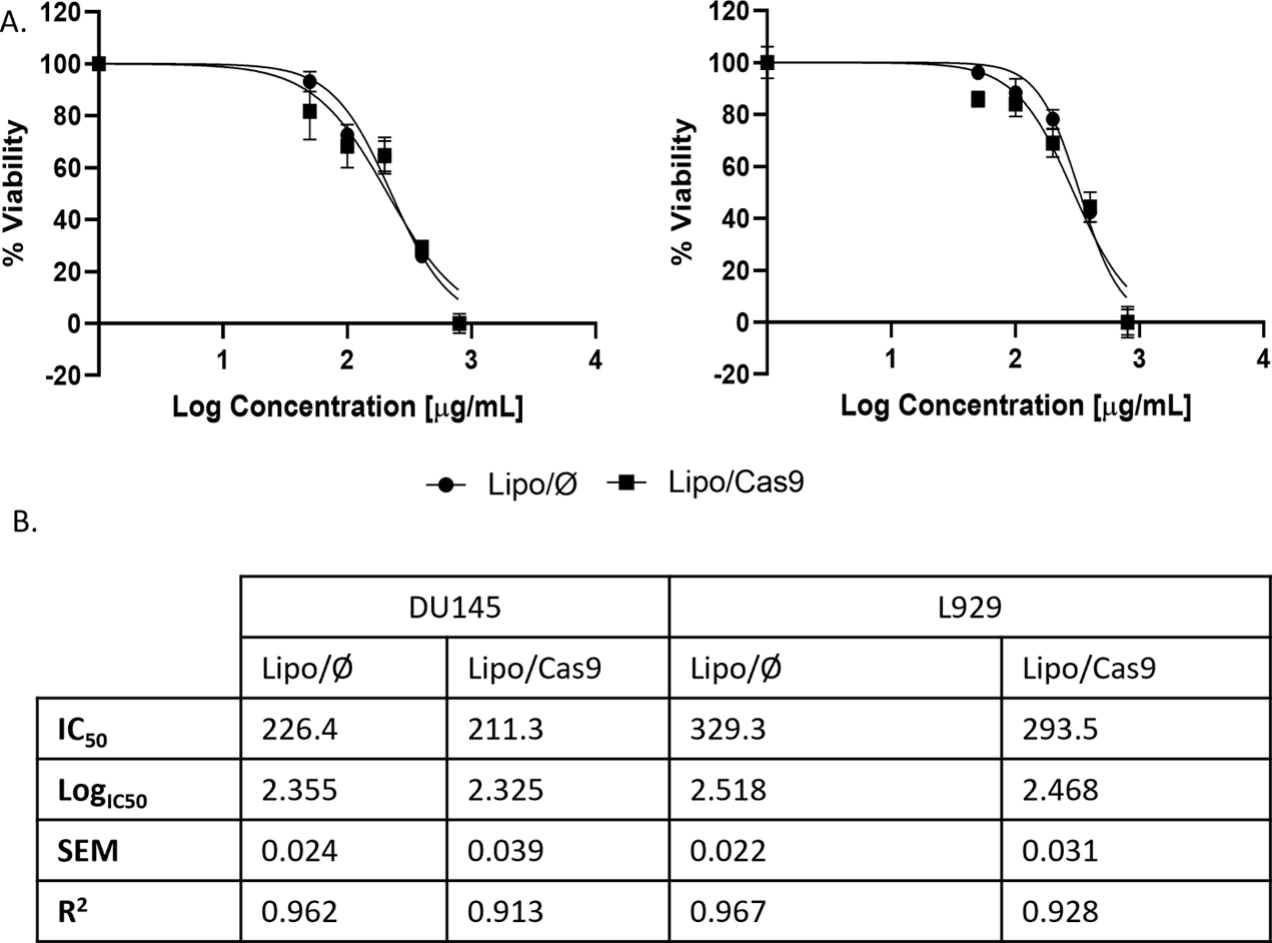
*In vitro* cytotoxicity and cellular uptake studies: (A) *in vitro* cytotoxicity graphs of Lipo/⌀ and Lipo/Cas9 on DU145 and L929cell lines (IC_50_ as μg/mL). (B) IC_50_, log IC_50_, and *R*^2^ values are given in a tabular form.

The cytotoxicity study results of Lipo/Ø or Lipo/Cas9 using L929 cells showed up to a dose of 100 μg/mL. There is no significant change in the viability of the Lipo/Ø and Lipo/Cas9 formulations. Further increasing the doses results in a decrease in the viability of Lipo/Ø and Lipo/Cas9. Thus, there is a dose-dependent cytotoxicity caused by the formulation (Figure 4A). For the Lipo/Cas9 formulation, the IC_50_ values were 211.3 μg/mL for the DU145 cell line and 293.5 μg/mL for the L929 cell line. Likewise, the values were determined as 226.4 and 329.3 μg/mL for the Lipo/Ø formulation, respectively. There was no significant difference analyzed in the viabilities of Lipo/Ø and Lipo/Cas9. However, slightly but not significantly higher cytotoxicity was observed on the DU145 cell compared to the L929 cell for both Lipo/Ø and Lipo/Cas9 formulations (Figure 4B).

**Figure 5:**
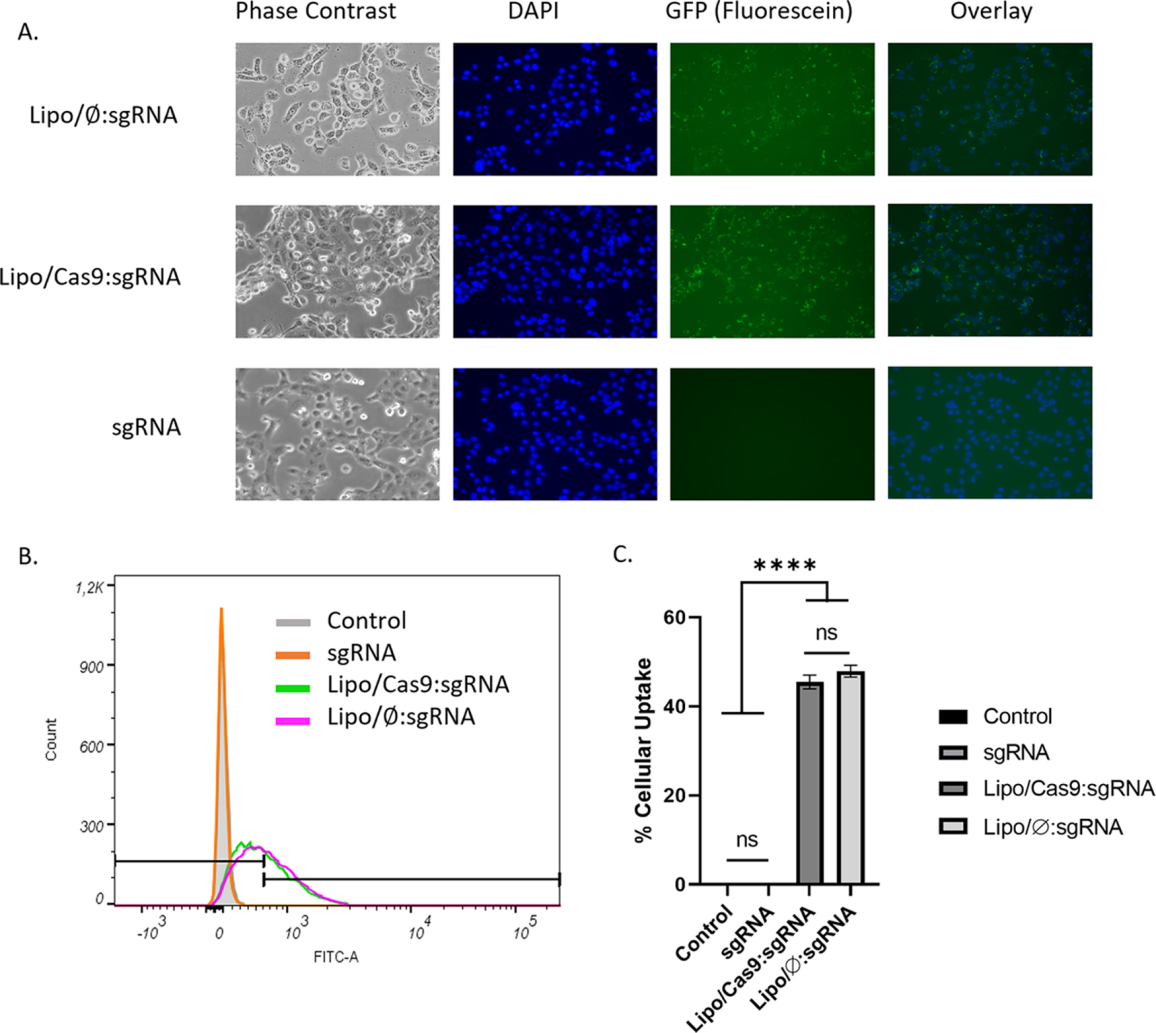
*In vitro* cytotoxicity and cellular uptake studies: (A) representative fluorescence microscopy images of naked sgRNA, Lipo/⌀:sgRNA, and Lipo/Cas9:sgRNA (1.5:1) groups for the qualitative analysis of *in vitro* cellular uptake efficiency (respectively from left to right, phase contrast images, DAPI-stained cells, images of fluorescently labeled sgRNA in cells under a fluorescence microscope, and the overlay images). (B): Representative flow cytometry histograms of naked sgRNA, Lipo/⌀:sgRNA, and Lipo/Cas9:sgRNA (1.5:1) groups. (C): Quantitative analysis of fluorescent dye percentage in living DU145 cells by flow cytometry analysis (**** = *P* < 0.0001, ns: not significant). Data are presented as mean values ± SD of triplicate treatments (*n* = 3).

*In vitro* cellular uptake study was performed using the naked sgRNA, Lipo/⌀:sgRNA, and Lipo/Cas9:sgRNA (1.5:1, N^+^/P^–^) groups with fluorescently labeled sgRNA on the DU145 cell line. The uptake efficiency was evaluated both qualitatively and quantitatively. Figure 5A shows the images of threatened cells, and a fluorescent dye was observed with inverted fluorescent microscopy. Lipo/⌀:sgRNA and Lipo/Cas9:sgRNA formulations showed similar fluorescence, while no fluorescence was detected in naked sgRNA. In order to demonstrate the cellular uptake efficiency quantitatively, flow cytometry was performed under the blue laser. The highest cellular uptake was obtained for Lipo/⌀:sgRNA and Lipo/Cas9:sgRNA with no significant difference in between, measured as 47.9 and 45.6%, respectively. Naked sgRNA has a shallow level of cellular uptake, as measured at 0.3%, and no significant fluorescence signal was observed for control cells (Figure 5B).

Gene expression levels were assessed following the incubation of the treated DU145 cells for 48 h at 37 °C. Lipo/⌀, Lipo/Cas9 formulations, and Lipo/Cas9:sgRNA (1.5:1) complexes with various sgRNA designs were evaluated by RT-qPCR. Expression of the SRD5α2 mRNA level was investigated according to reference GAPDH gene expression.

**Figure 6:**
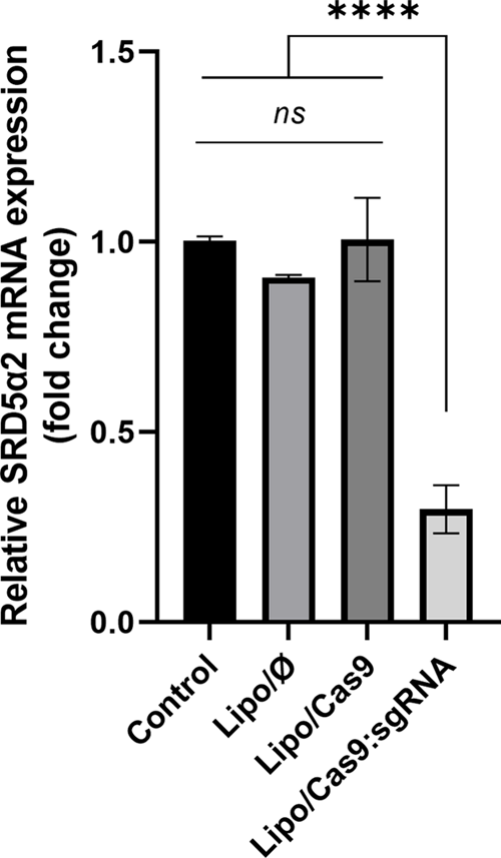
Expression folds change values and statistical evaluation of 5-alpha-reductase mRNA expression in the DU145 cell line according to the reference GAPDH gene expression (** = *p* < 0.01. **** = *p* < 0.0001. ns: not significant).

Lipo/Cas9:sgRNA (1.5:1) formulation that complexed with sgRNA decreased the relative mRNA expression by 29.7% compared to that of the control (Figure 6). The sgRNA design was carried out through the online Chopchop system. *In silico* analyses ranked the designed sgRNA as rank one due to its highest efficiency and lowest off-targeting properties. The designed sgRNA IDT was checked with the CRISPR-Cas9 guide RNA design checker application; the on-target score was found to be 29, and the off-target score was found to be 95. In terms of Lipo/⌀ and Lipo/Cas9 formulations, there was no significant change detected in the relative mRNA level for the bare Lipo/⌀ and Lipo/Cas9 formulations as in the control group, whereas the formulations had no effect on gene editing.

## 4 Discussion

Liposome formulations with DOTAP exhibit a range of particle sizes and distributions, depending on the composition and preparation method. Park et al. reported that particle size and zeta potential of liposomes varied depending on the ratio of DOTAP/DOPE/CH in the range of 270–350 nm and 0.8–9.7 mV, respectively.^41^ Optimization studies by Haghiralsadat et al. found that the liposomal cationic lipid, DOTAP, in combination with stearoyl phosphoethanolamine-polyethylene glycol, produced stable round-shaped particles without the aggregate formation and an average diameter of 71 nm.^43^ DOTAP/cholesterol liposomes could also resist destabilizing effects from serum proteins.^44,45^ The protein delivery efficiency of liposomal formulations would depend on the average diameter and the cellular uptake. Yan and Huang demonstrated that 20 μg of ovalbumin (OVA) formulated in 200 nmol DOTAP with a particle size range of 350–550 nm, protein loading capacity of 95–90%, and zeta potential of 29–38 mV had the best OVA-specific antibody response, suggesting both Th1 and Th2 immune responses were generated by this formulation.^46^ Employing DOTAP liposomes for gene therapy, Vemana et al. found that nanosized liposomes demonstrated desired transfection efficiency, negligible hemolysis, and minimal cytotoxicity.^47^ Although varying in particle size and distribution, liposome formulations with DOTAP generally offer excellent potential for protein, drug, and gene delivery.

The liposome preformulations and final formulations obtained within the scope of this study are in accordance with the general liposome characteristics specified in the literature. The optimized final formulation Lipo/Cas9’s average diameter was 190.7 ± 0.56 nm, the polydispersity index was 0.160 ± 0.007, and the zeta potential value was 36.6 ± 1.25 mV. Although the Lipo/Cas9 formulation lost its stability on day 60, the Lipo/⌀ formulation remained stable throughout the monitoring period. It can be stated that the increase in the particle size may be caused by protein aggregation. One factor that contributes to this phenomenon is protein denaturation and oxidative reactions. Another factor that influences particle size increase is the adsorption behavior of proteins on nanoparticles.^48,49^ In order to determine the optimal complex ratio of Lipo/Cas9 and sgRNA, a gel retardation assay was performed, and a 1.5:1 N^+^/P^–^ molar ratio was determined as the optimal complex ratio for sgRNA solution (110 μg/μL). A slight particle size increase was detected for the Lipo/Cas9:sgRNA complex (1.5:1) and measured as 229.1 ± 3.66 nm due to complex formation with sgRNA. Conversely, the zeta potential decreased to 25.7 ± 0.87 mV due to the negative charge of the sgRNA as expected.

The particle size distribution of liposome formulations plays a crucial role in determining their efficacy. Monodispersity is an essential characteristic in the preparation of liposomes, as it affects the encapsulation efficiency and stability. Most references show that the prepared liposomes have a narrow size range and low polydispersity index, indicating the formation of highly monodisperse liposome populations. The values of PI lower than 0.3 of liposomal dispersion can be considered homogeneous and monodisperse.^50,51^ The PI of all formulations produced in this study, including the preformulations, was below 0.3 and was determined to be 0.089 ± 0.013 for the final Lipo/Cas9:sgRNA complex (1.5:1). All of the formulations are monodispersed. The results obtained overlap with the results of the morphological examination with SEM and provided a correlation in terms of the physicochemical properties.

A serum stability test was conducted to analyze the Lipo/Cas9:sgRNA complex (1.5:1) system’s steric protection against serum proteins and nucleases. The structure of the complex is an essential parameter for the stable delivery of the nucleic acids and release into the cytoplasm at the endosomal pH after entering the cell.^52^ After systemic administration, sgRNA encounters many nucleases in the extracellular fluid, intercellular fluid, and circulation. RNA stability in liposome formulations plays a significant role in their effectiveness as delivery agents. Pereira et al. demonstrated a complexation stability of over 82% between DOTAP-DOPE liposomes and oligonucleotides during a 24 h native human serum exposure.^53^ The stability of cholesterol-rich liposomes was found to be higher than that of cholesterol-poor ones, leading to a more stable liposome structure.^54,55^ Accordingly, it was demonstrated by agarose gel electrophoresis that the Lipo/Cas9:sgRNA complex (1.5:1) preserved sgRNA for up to 3 h in the medium containing 10% serum and up to 1 h in the medium containing 50% serum (Figure 2C).

The EE of Cas9 RNPs in DOTAP liposome formulations varies, depending on the specific study and formulation conditions. In a study by Haghiralsadat et al., the optimized DOTAP liposome formulation had an EE of 89%.^43^ Similarly, Hiray and Krishnan and Hiray reported an encapsulation efficiency of 97.5 ± 0.8% for DOTAP liposomes.^56^ Other studies have also investigated the EE of cationic liposomes prepared from DOTAP and found around 80%.^57,58^ The release profile of Cas9 RNPs from delivery systems is crucial for their functionality and EE. Studies have shown that the release of Cas9 RNPs is more favorable under acidic conditions, similar to the endosomal environment, with a higher percentage released at pH 5 compared to those at pH 6 and pH 7.4.^59^ Direct delivery of Cas9 RNPs has advantages over plasmid or mRNA delivery methods, including reduced off-target effects, low toxicity, and high editing efficiency.^61,70^ To ensure effective genome editing, it is critical for Cas9 RNPs to be timely released in the cytosol and enter the nucleus after cell internalization.^61^ In this context, the EE and LC of the optimal formulation were investigated. Accordingly, the EE was determined to be 80.6% and the LC was determined to be 12.59%. The release profile of the Cas9 enzyme has been investigated, and it was revealed that the Lipo/Cas9 formulation was able to release a large extent of the Cas9 enzyme within 1 h, in accordance with the literature. The release of Cas9 enzyme from the Lipo/Cas9 formulation follows the burst release profile. After this burst release, it is followed by a continuous release for a certain period of time in accordance with the nanoformulation structure. This may be due to the composition of the nanoparticles, the protein–lipid interaction, and the protein adsorption on the surface of the nanoparticles.^62,63^

A dose-dependent cytotoxicity was observed in both DU145 and L929 cell lines due to the formulation ingredients. In both cell lines, the obtained results were parallel to the preformulation, and there was no significant difference between the Lipo/⌀ and Lipo/Cas9 formulations. Based on cytotoxicity data, doses that did not show relatively significant cytotoxicity were used in *in vitro* efficacy studies.

Dutasteride inhibits both isoenzymes of 5-alpha reductase (types 1 and 2), while finasteride inhibits only 5-alpha-reductase type 2. Studies have shown that dutasteride is a more potent inhibitor of 5-alpha-reductase compared to finasteride. The clinical potential of these inhibitors is indicated according to the extent to which they reduce the conversion of testosterone to DHT by inhibiting 5-alpha reductases. Finasteride has a variable half-life of 6–8 h, reducing the level of DHT by 70% when used at a 5 mg/day dose.^64^ Dutasteride inhibits 5α-reductases, leading to a 95% decrease in serum DHT concentrations.^65^ However, it has been reported that long-term inhibitor use also causes upregulation of 5-alpha reductase.^66,67^ Therefore, the genetic knockout of 5-alpha reductase maintains its clinical potential.

The delivery of Cas9 ribonucleoproteins (RNPs) into cells has been investigated using various methods such as nucleofection, electroporation, chemical transfection, and extracellular vesicles (EVs).^58,68−70^ These methods have shown varying efficiencies, in terms of uptake. In a study, nucleofection of Cas9 RNPs into HEK293T cells resulted in knockout frequencies of around 10%, with higher efficiencies observed in synchronized cells.^58^ Higher knockout results obtained in HEK293 cells using lipoplexes showed efficient VEGFA knockout (43% indels) and GFP knockout (approximately 28%) in various studies.^71,72^ RNP-based delivery of CRISPR/Cas9 showed good efficiency of genomic rearrangements in human hematopoietic stem and progenitor cells.^73^ Additionally, viral glycoproteins and engineered EVs have been explored to enhance the delivery efficiency of Cas9 RNPs.^69,74^ A cellular uptake of over 50% has been reported in a study using poly sgRNA/siRNA RNP nanoparticles for targeted gene disruption.^75^ Overall, the uptake efficiency of Cas9 RNPs varies depending on the cell type and the delivery method employed. Hereby, a high level (∼50%) of cellular uptake was determined for the Lipo/⌀:sgRNA and Lipo/Cas9:sgRNA complex (1.5:1) according to the quantitative analysis of cellular uptake studies (Figure 5B). After the cytotoxicity and cellular uptake studies, gene knockdown studies were carried out. In this context, gene knockout in the DU145 cell line was investigated by RT-qPCR to determine the SRD5α2 expression change at the mRNA level after administration. Accordingly, the Lipo/Cas9:sgRNA (1.5:1) formulation complexed with sgRNA decreased the relative mRNA expression by 29.7% compared to the control group. For Cas9-mediated gene knockout with RNP transfection, it has been shown that the knockout efficiency ranges from 15 to 90% in various cell types.^76^ Similarly, in mouse bona fide hematopoietic stem cells (HSCs), the knockout efficiency of integrin alpha 2b (Itga2b) using Cas9/RNP was estimated to be approximately 15%.^77^ In human HEK293 cells, the knockout efficiencies achieved with Cas9/gRNA were 10–25%.^26,70^ Higher gene knockout levels were also obtained depending on the cell type and delivery method. In a study, a light-sensitive liposome delivery system was developed for gene editing with CRISPR/Cas9, demonstrating a high transfection efficiency with knockout percentages of 52.8% in HEK293 cells.^78^ Other factors, such as the sgRNA design and the expression level of Cas9 and delivery methods, can also affect the knockout efficiency.

## 5 Conclusions

BPH, PC, and androgenic alopecia (AA) are three conditions associated with androgen activity and the 5-alpha-reductase enzyme. 5-Alpha-reductase inhibitors have been used for the treatment of BPH, PC, and AA. Considering their side effects, an alternative treatment approach to 5-alpha-reductase enzyme inhibitors has been developed due to their essential role in steroid mechanisms. Contrary to the drugs used without disturbing the steroid metabolism of the organism, with a single dose application, the inhibition of testosterone metabolism in the desired region by inhibiting the conversion of testosterone to DHT was achieved via genetic knockout of the dominant SRD5α2 isozyme of 5-alpha reductase. This treatment approach could reduce the undesirable effects of DHT without changing the serum testosterone level.

This study developed a hybrid delivery system to provide gene knockout of SRD5α2 in DU145 cells. This system also provides sgRNA variability as the sgRNA is transferred to the cell separately on the surface of the liposome formulation while performing Cas9 enzyme immobilization in liposomes. No Cas9 enzyme immobilization and delivery approach by liposome formulation designed in this way has been found in the literature. While this therapy approach holds promise for treating PC, there are still challenges to overcome including targeting the therapeutic genes to target cells and ensuring higher gene knockout efficiency. Preliminary data have been obtained to develop a treatment approach that is free from the side effects of the drugs used by targeting or directly applying this system to the target tissue in further studies. The results obtained with this project will pave the way for different studies on effective gene therapy approaches against BPH, PC, or AA.

## Abbreviations

SRD5α2: steroid type II 5-alpha-reductase
DHT: dihydrotestosterone
CRISPR: clustered regularly interspaced short palindromic repeats
Cas9: CRISPR-associated protein 9
BPH: benign prostatic hyperplasia
PC: prostate cancer
AA: androgenic alopecia
sgRNA: single guide RNA
hnRNPs: heterogeneous nuclear ribonucleoproteins
DOPE: 1,2-dioleoyl-*sn*-glycero-3-phosphoethanolamine
DSPE-PEG_(2000)_-DBCO: 1,2-distearoyl-*sn*-glycero-3-phosphoethanolamine-*N*-[dibenzocyclooctyl(polyethylene glycol)-2000]
DOTAP: 1,2-dioleoyl-3-trimethylammoniumpropane
AD: average diameter
PI: polydispersity index
ZP: zeta potential
SEM: scanning electron microscope
DLS: dynamic light scattering
DMEM: Dulbecco’s modified Eagle’s medium
